# Specialized diving traits in the generalist morphology of *Fulica* (Aves, Rallidae)

**DOI:** 10.1038/s41598-024-64853-4

**Published:** 2024-06-17

**Authors:** Ricardo Santiago De Mendoza, Julieta Carril, Federico Javier Degrange, Claudia Patricia Tambussi

**Affiliations:** 1 Laboratorio de Histología y Embriología Descriptiva, Experimental y Comparada (LHYEDEC), Facultad de Ciencias Veterinarias, Universidad Nacional de La Plata, Consejo Nacional de Investigaciones Científicas y Técnicas (CONICET), Buenos Aires, Argentina; 2 Centro de Investigaciones en Ciencias de La Tierra (CICTERRA), Universidad Nacional de Córdoba, Consejo Nacional de Investigaciones Científicas y Técnicas (CONICET), Córdoba, Argentina

**Keywords:** Water birds, Coots, Disparity, Ecomorphology, Swimming birds, Generalist taxa, Palaeontology, Herpetology

## Abstract

Foot-propelled diving comprises the primary locomotion-based feeding strategy for many birds, including families such as Phalacrocoracidae, Anhingidae, Podicipedidae, Gaviidae, and the diving ducks within Anatidae. While the morphology of specialized divers is well known, the corresponding morphology is less known for birds not as specialized but capable of diving, such as the coots (Rallidae, *Fulica* spp.). To compare the osteology of *Fulica* with other (non-diving) Rallidae, and with foot-propelled diving birds that are distantly related, we considered osteological characters, as well as the proportion of the hind limb bones and the femoral splay angle to construct a phylomorphospace, and to perform a comparative disparity analysis considering ecomorphologically relevant characters related to swimming and diving. Coots resulted to be significantly disparate from other Rallidae showing many traits of specialized foot-propelled divers, but only noticeable when compared with other rallids, as the degree of development of these traits is markedly less than in loons, grebes, or cormorants. This may correspond to a stabilizing selection of characteristics associated with a generalist morphology in *Fulica*. Studying adaptation in generalist taxa broadens our understanding of ecomorphologically significant features, thereby enabling us to generalize their evolutionary patterns.

## Introduction

Diving is a widespread skill among tetrapods that can be performed in various scenarios such as escaping from a predator or acquiring food^1^. In birds, underwater propulsion can be facilitated by either wings (wing-propelled divers like penguins and alcids) or legs (foot-propelled divers like cormorants or ducks)^1^. The body shape, pelvis, limbs, digits, and their associated musculature are quite distinctive in divers^2^. The body is elliptical in shape, with narrow sternum and pelvis^2–4^, and the hind limbs are located very posteriorly (in the most extreme cases like in grebes, hind limbs almost align with the main axis of the body)^5^. The femur is short and craniocaudally curved, the crista cnemialis cranialis of the tibiotarsus is long, the feet are palmate or totipalmate^5^, and the feathers are arranged very close to the body, thereby reducing friction during underwater locomotion^6^.

A number of extinct birds, such as the Cretaceous *Hesperornis* and *Vegavis*, as well as some modern birds, are considered to be foot-propelled divers^7–9^. Among extant birds, foot-propelled divers mainly include cormorants, shags, darters, loons, grebes, and certain diving ducks^9^. None of these major groups of divers are closely related to each other^9^, suggesting that foot-propelled diving primary lifestyle is likely a result of convergent evolution.

Many birds, like ducks, phalaropes and most foot-propelled diving birds, are buoyant swimmers^1^. In birds that dive, this is because its plumage remains dry after diving^10^. However, this is not the case for cormorants and darters^10^. When these taxa swim on the surface, they are partially submerged, with only their head and neck emerging. This is because they have “partially wettable” plumage^10^. Both can feed by picking items from the surface of the water, but only the former can upend their bodies and feed by dabbling^1^.

While the morphology of specialized divers is well known^2,5,7^, the knowledge on the morphology of birds not specialized but capable of diving is still scarce. For instance, coots (Rallidae, *Fulica* spp.) appear to be generalists in terms of their body shape, but quite specialized in terms of their locomotor skills^11^. Coots have strong legs and large, lobed toes. Their wings are short and when taking off from the water they need to move them strongly, hitting the surface^11^. Coots are capable swimmers and divers with specialized flexors and extensors of the tarsometatarsus and digit 3 during diving^12^. To submerge, they deploy an arc with their body out of the water, bring their wings closer to their body and when their feet are completely out, they submerge their head and jump^13^.

Rallidae is a clade of Gruiformes that comprises 155 species across 37 genera^11^, with a widespread distribution including all the continents except Antarctica. They inhabit a diverse range of environments and exhibit a variety of locomotor behaviors. Some species, such as those within *Rallus*, *Laterallus*, and *Pardirallus* genera, primarily reside in densely vegetated marshes and mangroves^11,14,15^. Others, like *Gallinula*, are more aquatic, frequently swim on the surface occasionally dabbling or upending their bodies (a few species even dive on rare occasions)^11^. In contrast, most species of *Fulica* are highly aquatic and often dive^14,15^. Particularly, *Fulica armillata* is a frequent and skilled diver^16^.

Here, we will concentrate on examining the skeleton of the rallids, with focus on the coots *Fulica* in the context of diving birds, as they are foot-propelled divers that have an atypical body shape. The aims of this study are: (1) to examine the morphological and morphometric characteristics of the skeleton of the coots within a comparative context of diving birds, and (2) to determine whether coots are significantly disparate from other rallids in terms of morphological and morphometric features associated with diving behavior. For these, we apply morphometric techniques, construct a phylomorphospace, and perform a morphofunctional disparity analysis.

## Results

### Sternum width and rib orientation

**Figure 1:**
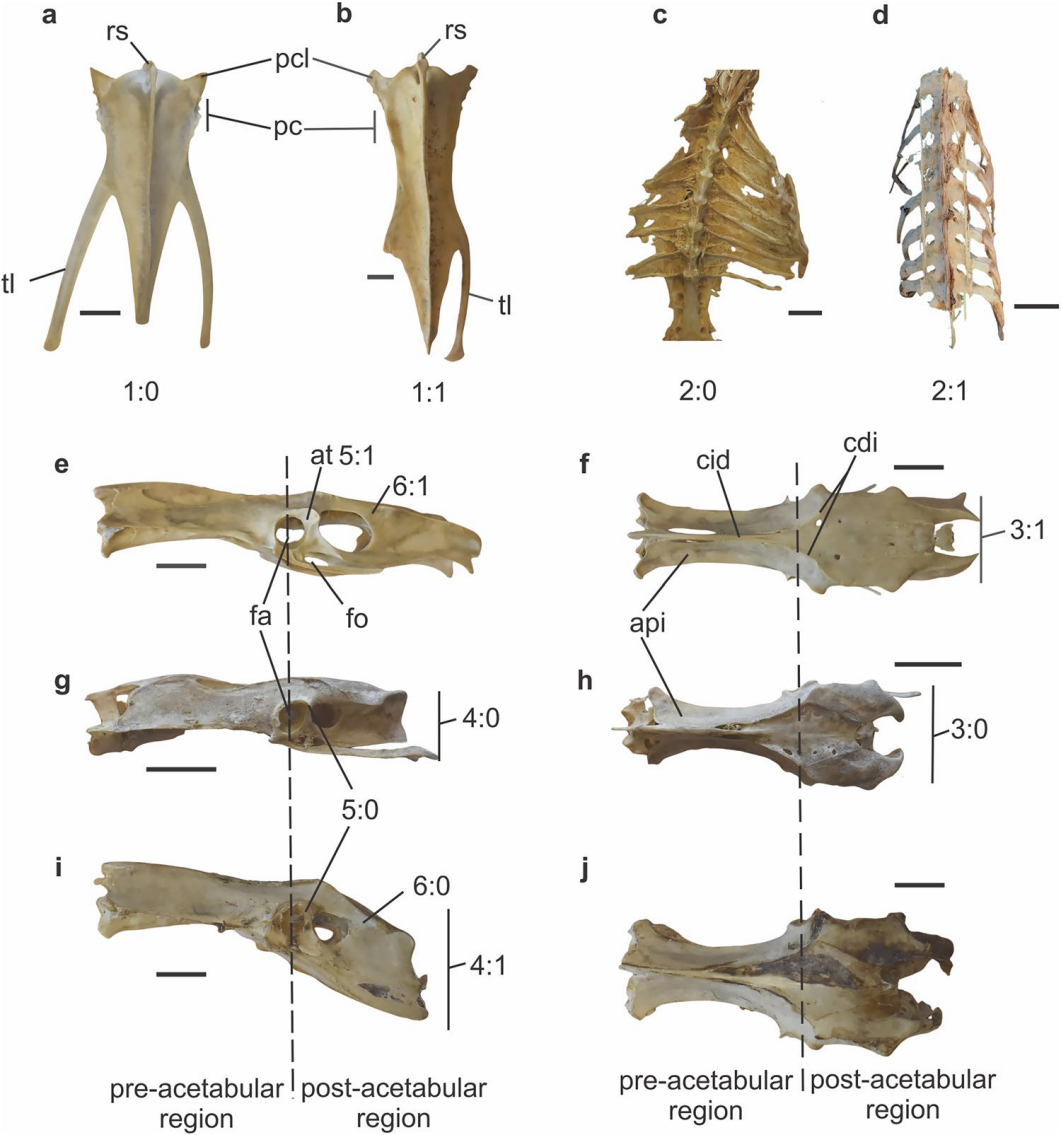
Sternum of (**a**) *Fulica leucoptera* CIT-O 216; (**b**) *Aramides* sp. CIT-O 14. Character states signaled on the bones. Ribcage of (**c**) *Fulica leucoptera* CIT-O 50; (**d**) *Porphyrio martinica* CIT-O 148. (**e**–**j**) Pelvis of Rallidae. (**e**,**f)** *Fulica leucoptera* CIT-O 216; (**g**,**h**) *Pardirallus maculatus* CIT-O 151; (**i**,**j**) *Porphyrio martinica* CIT-O 148. (**e**,**g**,**i**), lateral view; (**f**,**h**,**j**), dorsal view. Character states signaled on the bones. *fa* foramen acetabularis, *fo* foramen obturatum, *at* antitrochanter, *cid* crista iliaca dorsalis, *cdi* crista dorsolateralis ilii, *api* ala preacetabularis ilii, *pc* processus costalis, *pcl* processus craniolateralis, *rs* rostrum sterni, *tl* trabecula lateralis. Scale bars: 10 mm.

In general, and unlike most non-diving birds, rallids have a surprisingly compressed body, with narrow sterni (Fig. 1a,b) and ribs oriented ~ 45° caudally (Fig. 1d), as seen in the swamphen *Porphyrio*. Nonetheless, this is not the case of *Fulica* in which the ribs, particularly those pairs located in the caudal half of the ribcage, contact in higher angles or are even perpendicular with the spine (Fig. 1c). Additionally, the sternum in *Fulica* is wider than that of other rallids.

The sternum in *Fulica* (Fig. 1a) has pointed processi craniolaterales, which are further apart from the rostrum sterni compared to *Porphyrio* or *Aramides*, and closer in position to those in ducks. Also, the space between the caudalmost processi costales and the trabeculae laterales is less than half in *Fulica* than in other rallids. The trabecula lateralis is straight and points laterocaudally, clearly extending beyond the caudal end of the margo caudalis. In other rallids, the trabecula lateralis curves caudally, aligning with the pointy margo caudalis and slightly surpassing it. The shape of the sternum is also shared with *Gallinula*^17^. The more lateral oriented trabecula lateralis and wider cranial margin result in a broader sternum in *Fulica* and *Gallinula* compared to other rallids. This, combined with the larger angles between the ribs and the spine, creates a wider profile.

### Forelimb

Diving birds usually have relatively short wings^18^, which reduces the drag and thus facilitate diving. Nonetheless, this is not an exclusive feature of divers^18^. In fact, all non-diving rallids have short wings^19^. The relative size and proportion of the elements is about the same in all rallids. *Fulica* shows a slenderer humerus than *Aramides* but not than *Pardirallus* or *Porphyrio*, and a shorter carpometacarpus than these taxa but not than *Pardirallus*.

### Pelvis

A narrow pelvis is characteristic of diving taxa such as cormorants, loons, grebes, and auks^2,5^ among others. All rallids have narrow pelvi^15^, although not as narrow as cormorants, but narrower than diving ducks. In all rallids, the alae preacetabulares ilii, when viewed dorsally, are very narrow in the middle of the pre-acetabular region, being broader at the cranial rim of the foramen acetabuli, giving a shape like a pair of outward-facing parentheses to the preacetabular region (Fig. 1e–j). In *Aramides* and *Porphyrio*, these ‘parentheses’ are more rounded, while they are straighter in *Pardirallus*, and especially in *Fulica*. In all rallids, the cristae iliaca dorsales run along the midline of the crista spinosa synsacri, forming a single narrow edge. About halfway along the pre-acetabular region, the crista iliaca dorsalis divides into the cristae dorsolaterales ilii. However, this division occurs more caudally in *Fulica*, just cranial to the acetabula. This difference may also be related to the presence of a narrower pelvis, especially in the dorsal part. The distance between the acetabula is smaller in *Fulica* than in the rest^15,17^. The dorsal part of the ischia is wider than the space between the caudal rims of the acetabula in all rallids except for *Pardirallus* and *Fulica*, where it has the same size or it is slightly smaller. This coincides with the narrower pelvis common in diving birds.

The post-acetabular region is longer in *Fulica* than in other rallids^15^. The acetabulum is positioned in the middle of the entire structure, similar to ducks or cormorants and unlike other rallids. In all examined rallids, except *Fulica* and *Pardirallus*, the caudal part of the ischium is wider than the cranial part in the dorsoventral direction. In *Fulica* and *Pardirallus* the ischium is not dorsoventrally wider in the caudal-most part, like in diving taxa. As a result, the longer axes of the foramen obturatum and fenestra ischiopubica are parallel to the longer axis of the ilium. In contrast, in other rallids, the longer axes of both foramen and fenestra are angled with respect to the longer axis of the ilium.

The antitrochanter is well marked in *Fulica*, more than in the other rallids. Its articular surface with the fascies articularis antitrochanterica of the femur is more visible in lateral view than in the rest of rallids. However, the antitrochanter angle is about the same as in the other analysed rallids.

### Femur

**Figure 2:**
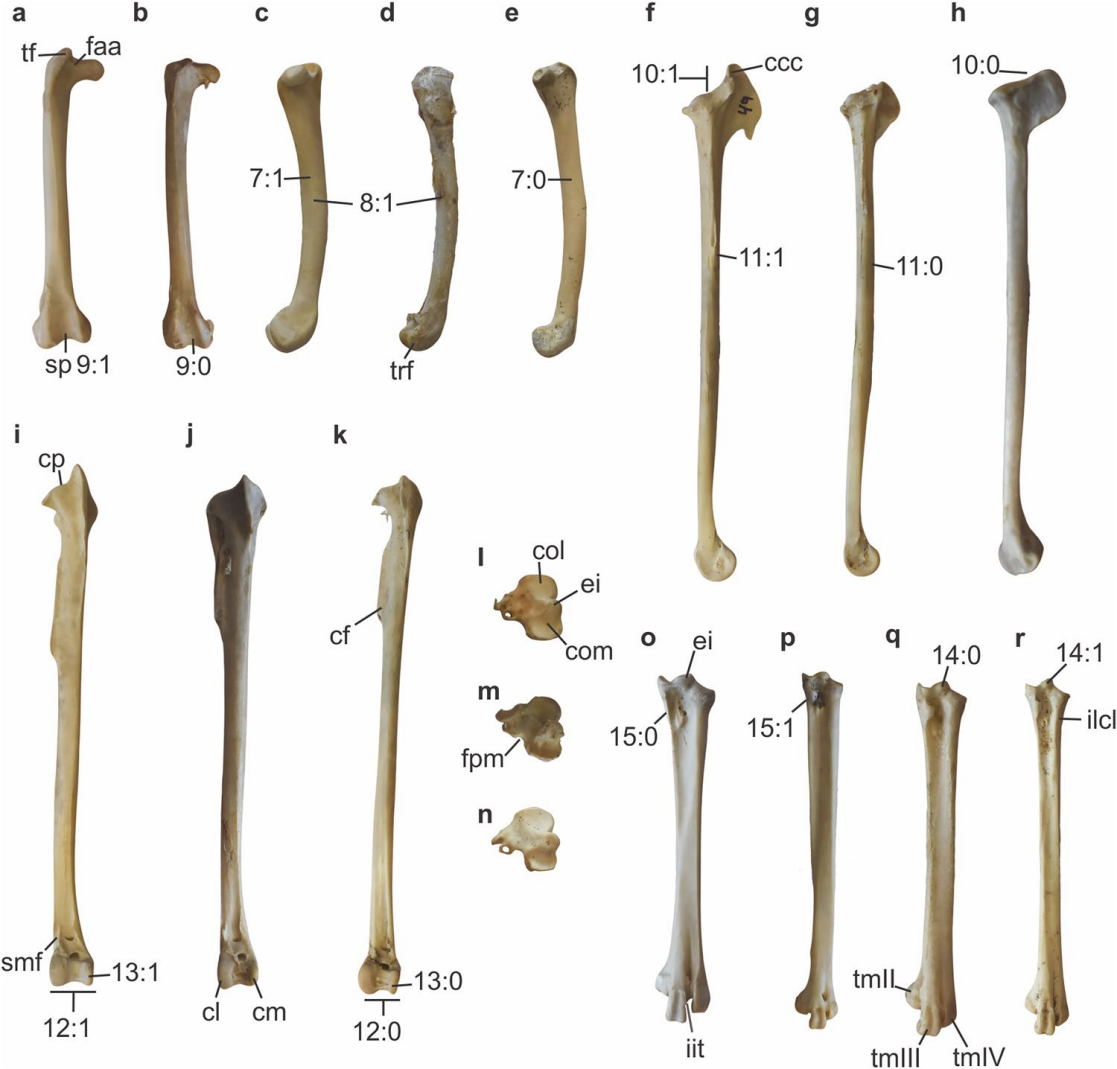
Hind limb bones of Rallidae. (**a**–**e**) femur; (**f**–**k**) tibiotarsus; (**l**–**r**) tarsometatarsus. (**a**,**c**,**f**,**i**) *Fulica armillata* CIT-O 49; (**b**,**d**,**g**,**m**,**p**) *Porphyrio martinica* CIT-O 148; (**e**,**k**,**n**,**r**) *Aramides* sp. CIT-O 14; (**h**,**j**,**k**) *Pardirallus maculatus* CIT-O 151; (**l**,**o**) *Fulica leucoptera* CIT-O 216. (**a**,**b**,**i**–**k**) cranial aspect; (**c**,**e**) medial view; (**d**,**f**–**h**) lateral aspect; (**l**–**n**) proximal end; (**o**–**r**) dorsal aspect. Character states pointed on the bones. *ccc* crista cnemialis cranialis, *cf* crista fibularis, *cl* condylus lateralis, *cm* condylus medialis, *col* cotyla lateralis, *com* cotyla medialis, *cp* crista patellaris, *ei* eminentia intercotylaris, *faa* facies articularis antitrochanterica, *fpm* fossa parahypotarsalis medialis, *iit* incisura intertrochlearis medialis, *ilcl* impression ligamentum collateralis lateralis, *smf* sulcus musculus fibularis, *sp* sulcus patellaris, *tf* trochanter femoris, *tmII–IV* trochlea metatarsi II–IV, *trf* trochlea fibularis. Scale bars: 10 mm.

One of the best-known features shared by foot-propelled diving birds is the presence of a robust, craniocaudally bowed, and oftenly short femur^5,7,8,20,21^. This is the case for grebes, cormorants, and loons^5^. While the femur of diving ducks such as *Aythya*, *Somateria*, or *Oxyura* is curved, it is not as short and robust as in the birds mentioned above. In contrast, the diving duck *Biziura* has a shorter femur than other diving ducks^20^. In the case of the analysed rallids, the femur is slightly craniocaudally bowed, even in the most terrestrial species. This is also true for *Fulica*. On the other hand, in *Fulica* the femur (Fig. 2a) is more robust and has a shorter shaft than in other rallids. As a result, it is similar to the femur of shags in lateral view.

The trochanter femoris in *Fulica* is well-developed, elevated and larger than other rallids. Most foot-propelled diving birds, such as grebes, loons, and diving ducks, lack the elevation of the trochanter femoris^21,22^. As a result, the proximal end lateral to the facies articularis antitrochanterica has the same dorsoventral depth as the capitis femoris. Nonetheless, among foot-propelled diving birds, darters, cormorants, and shags have a well-developed and elevated trochanter femoris^23^.

At the distal end of the femur, the sulcus patellaris is broader in *Fulica* than in other rallids. The fossa poplitea in all rallids is shallow, and *Fulica* is not the exception. In diving birds, the trochlea fibularis is usually as large as the crista tibiofibularis^21^. However, in *Fulica* the trochlea fibularis is smaller than the crista tibiofibularis, as in most birds, including other rallids.

### Tibiotarsus

Foot-propelled divers typically exhibit an elongation of the crista cnemialis cranialis^21,24^. In *Fulica* (Fig. 2f), the crista cnemialis cranialis is slightly longer proximally than in other rallids^17^. However, it is much larger cranially and ends distally in a hook. It also extends as a sharp edge along the shaft of the tibiotarsus, reaching as far distally as the distal end of the crista fibularis, similar to diving ducks^21^. The crista patellaris in *Fulica* is proximally convex, whereas in other rallids it is concave.

As in foot-propelled divers^21^, *Fulica* have the facies cranialis flat, especially between the ridge distal to the crista cnemialis cranialis and the crista fibularis. In the rest of rallids, it is more rounded, as in most birds^21^.

In all rallids, in the distal end of the tibiotarsus, the condylus medialis is thinner than the condylus lateralis. However, the difference is smaller in *Fulica* than in other rallids. In *Fulica*, the lateral rim of the condylus lateralis in cranial aspect is aligned with the sulcus m. fibularis. In swimming taxa such as almost all anatids and diving taxa such as cormorants and loons, the lateral rim of the condylus lateralis is medial to the projection of the sulcus m. fibularis^25,26^. In all these taxa, the distal end of the tibiotarsus is located medially to the projection of the distal part of the shaft^25^. In contrast, in terrestrial taxa including rallids (aside from *Fulica*), and the extinct presbyornithids^25^ and the extant screamers^25,26^ among Anseriformes, the distal end of the tibiotarsus coincides with the projection of the tibiotarsal shaft. As a result, in these taxa the lateral rim of the condylus lateralis is lateral to the projection of the sulcus m. fibularis.

### Tarsometatarsus

In *Fulica*, the eminentia intercotylaris is proximally rounded and blunt (Fig. 2o). In dorsal aspect, it is less projected proximally than the medial rim of the cotyla medialis. In *Rallus*, it is more tappered and extends proximally at the same level as the medial rim of the cotyla medialis. In *Aramides* and *Porphyrio*, the eminentia intercotylaris is pointed, extending proximally beyond the medial rim of the cotyla medialis. Viewed proximally, the cotyla medialis is more rounded in *Fulica* than in the rest of rallids. In *Fulica*, in dorsal and plantar aspects, the area of the cotylae tapers to the area immediately below and tapers again below the impressio lig. collateralis lateralis (in the dorsal aspect) and the sulcus m. fibularis longus (in the plantar aspect). In *Porphyrio* and *Pardirallus*, this transition from the area of the cotylae to the shaft is almost straight, while in *Aramides* it is similar to *Fulica*.

In *Fulica* and *Gallinula*, the fossa parahypotarsalis medialis is absent, and the medial-most part of the hypotarsus merges smoothly with the plantar aspect of the shaft (Fig. 2l). On the other hand, in the remaining rallids, the fossa parahypotarsalis medialis is visible in proximal view as an indentation medial to the hypotarsus^27^.

*Fulica* has the shortest shaft relative to the size of its trochleae among all analysed rallids, being robust similarly to that of a duck. In contrast, *Pardirallus* has a relatively long and thin shaft. *Porphyrio* and *Aramides* have clearly elongated shafts. Notably, despite *Fulica* being a larger bird, the tarsometatarsus of *Aramides* is about twice as long.

In dorsal and plantal aspects, the configuration of the distal end and the position of the trochleae vary greatly among different diving and swimming taxa^28^. In *Hesperornis*, loons, and grebes, the trochlea metatarsi II ends more proximally than the incisura intertrochlearis lateralis, and the trochleae metatarsorum III and IV are approximately the same size^28^. In diving ducks, the trochlea metatarsi II also ends more proximally than the incisura intertrochlearis lateralis, but the trochlea metatarsi IV ends more proximally than the trochlea metatarsi III, as in other ducks^29^. This configuration is also present in some non-diving anatids, notably swans, but in most other anatids, the trochlea metatarsi II ends more distally than the incisura intertrochlearis lateralis^21,24,29^. On the other hand, in cormorants and darters, the trochlea metatarsi II is almost aligned with the trochlea metatarsi III and the trochlea metatarsi IV is variably smaller^23^. In rallids the configuration is very similar to that of ducks, and in *Fulica*, *Pardirallus* and *Aramides* the trochlea metatarsi II ends more proximally than the incisura intertrochlearis lateralis. The trochleae themselves are larger in *Fulica* than in the rest of rallids.

Viewed distally, the overall shape and orientation of the trochleae are similar in all rallids and closely resemble those of anatids^30^. In all cases, the lateral rim of the trochlea metatarsi IV is thin and sharper than the medial rim, and the trochlea metatarsi III is approximately symmetrical. The trochlea metatarsi II is asymmetrical, being the medial rim smaller dorsoplantarly than the lateral rim. In *Fulica*, both rims of the trochlea metatarsi II have a similar width, while in *Pardirallus*, *Porphyrio*, and *Aramides*, the medial rim is thinner.

### Ossa digitorum pedis

The lobate toes are arguably the most striking difference between coots and other rallids^11,28^. This external structure is shared with the closely related heliornithids^31^ and with the more distant phalaropes^32^. Olson^17^ reports incipient lobes in the feet of *Gallinula*. Similar structures are found in grebes^28^. A difference in size between the lateral and medial joints of the tarsometatarsus with the proximal phalanges, has been proposed as evidence of the presence of lobed toes^33^. However, this discrepancy in size is absent in *Fulica*^28^*.*

Large toes are a common feature of foot-propelled diving birds as a way to expand the propulsive surface^20,34^ but it is also present in wading birds^11^. *Fulica* has long toes on their feet^11^ as well as all rallids. The phalanges proximales et intermediae of *Fulica* are broader than those of *Porphyrio*, and the proximal part of the basis phalangis is bounded by sharp rims.

### Quantitative analyses

#### Femoral splay angle

**Figure 3:**
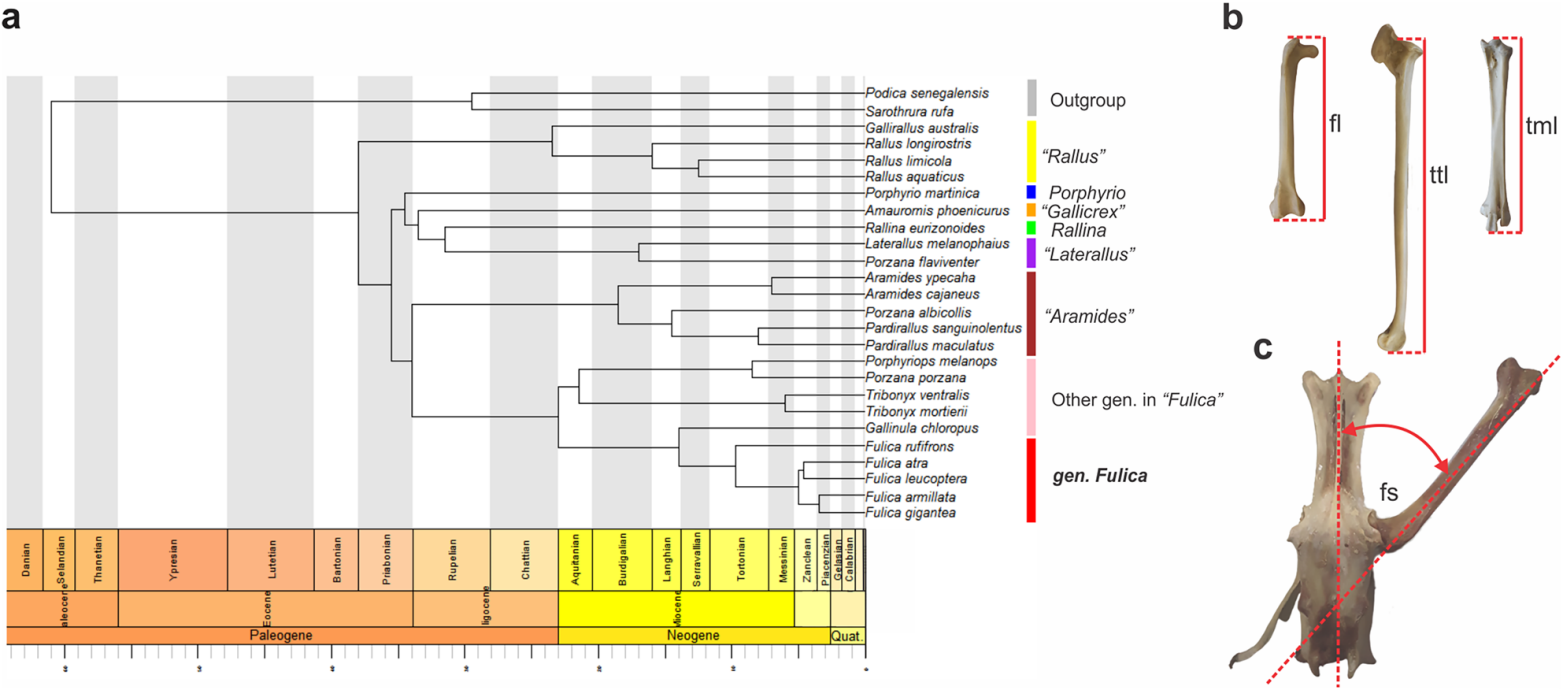
(**a**) calibrated phylogenetic tree of Rallidae based in García-R et al.^32^ including exclusively the taxa studied herein. (**b**) From left to right, measurements of the femur, tibiotarsus and tarsometatarsus maximum length; (**c**) femoral splay angle measurement. *fs* femoral splay angle, *fl* femoral length, *ttl* tibiotarsus length, *tml* tarsometatarsus length.

The femoral splay angle for the analysed rallids is listed in Supplementary Information S2 and displayed graphically in Fig. 3c. The variation found includes more than 20° among the species, ranging from 18° to 40°. The lowest values are overall found in *Aramides*, *Porphyrio*, *Gallinula* and *Porzana* (between 18 and 30°, with great variation in *Porphyrio*), while intermediate values are found in *Rallus*, *Laterallus* and *Pardirallus* (between 26° and 34°). The highest values are those of the two species of *Fulica* (between 30° to 40°).

**Figure 4:**
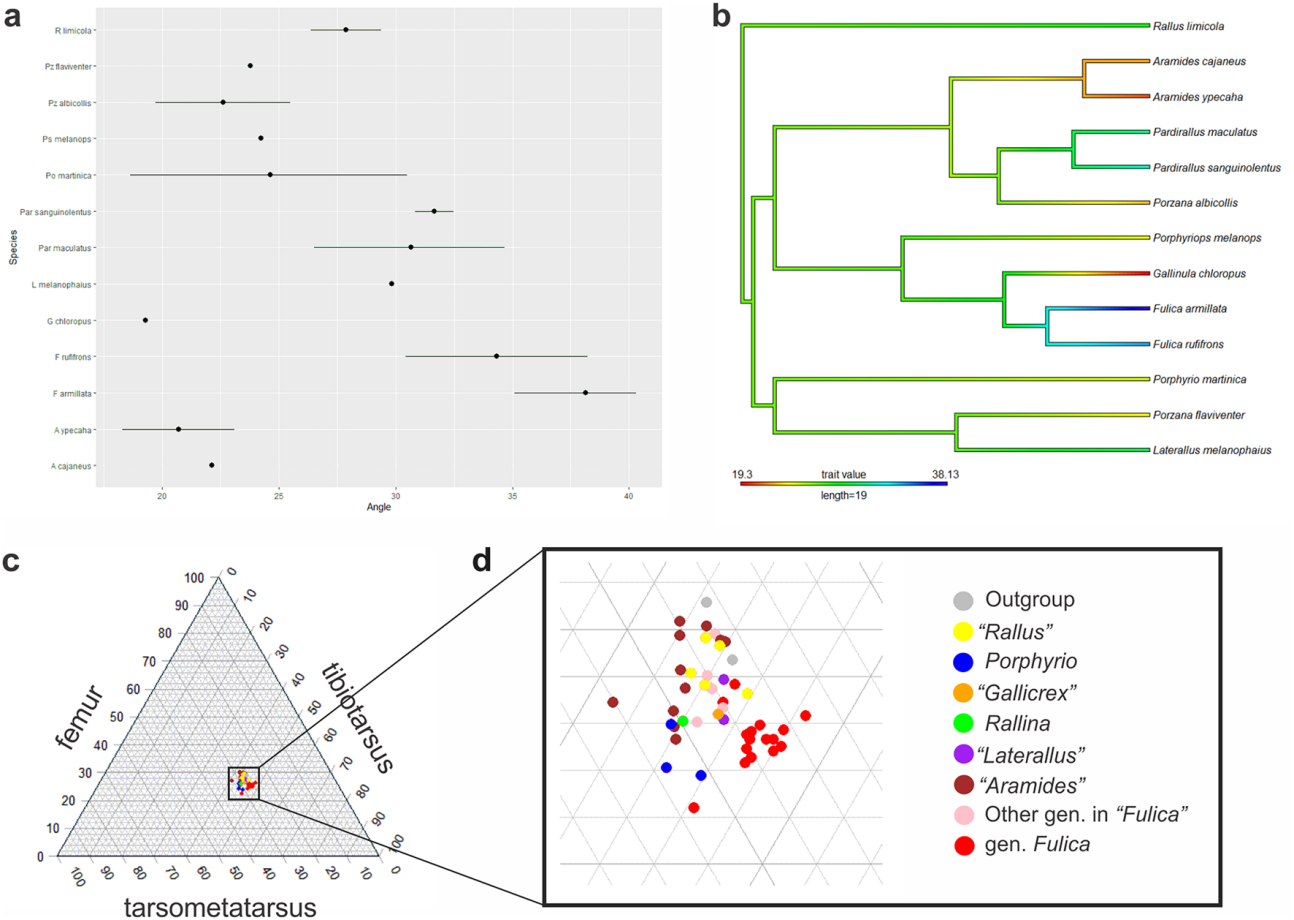
(**a**) point and range plot of the femoral splay angle for the analysed species. (**b**) Character mapping of femoral splay angle on the phylogeny of García-R et al.^32^ (see Fig. 3a,c). (**c**) Ternary plot of the relative length of femur, tibiotarsus and tarsometatarsus, colored according to belonging to clade within Rallidae (see Fig. 3a,b). The upper vertex of the plot corresponds to the highest proportion of the femur, while the lower left and lower right vertices correspond to the highest proportions of the tarsometatarsus and tibiotarsus, respectively. (**d**) zoom-in of the subfield of (**c**) where the analysed specimens are distributed.

The ancestor of all rallids had middle values for femoral splay angles (see character mapping in Fig. 4b). There are convergent decreases in the lines to *Porzana flaviventer* and *Porzana albicollis*, and a large decrease in *Aramides*. The mapping reveals middle values in the last common ancestor of *Porphyriops* + (*Gallinula* + *Fulica*), with a very small decrease in *Porphyriops melanops*. The most notorious is the large decrease in *Gallinula chloropus* while its sister group, *Fulica*, has a single (and strong) increase, especially in *Fulica armillata*.

#### Hind limb proportions

The raw measurements of the femur, tibiotarsus, and tarsometatarsus are shown in Supplementary Information S2. In the ternary plot (Fig. 4c,d), all the taxa are densely clustered together, occupying a continuous portion of the space. The distribution of dots is skewed away from the center towards an area where the tibiotarsus is proportionally longer (as it is the longest of the analysed hind limb bones across all examined taxa).

The two outgroup taxa are located in the upper part of the distribution, where the femur is proportionally longer, with Sarothruridae occupying the highest position. The taxa in the “*Aramides*” clade are primarily found in the upper and left portions of the plot, where the femur and tarsometatarsus are proportionally longer than in the rest of taxa. *Porphyrio* is situated in the lower left corner. *Rallina eurizonoides* is positioned very close to both the “*Aramides*” clade and *Porphyrio*. The clades “*Rallus*”, “*Laterallus*”, and “*Gallicrex*” are located around the middle of the plot, along with *Gallinula*, *Porphyriops*, and *Tribonyx*. These are the other genera, besides *Fulica*, that belong to the “*Fulica*” clade. Lastly, *Fulica* extends from the center to the lower right corner of the plot, indicating a proportionally longer tibiotarsus (as shown in Fig. 4d), which is a characteristic expected in swimming and diving taxa. Interspecific differences within *Fulica* are not readily apparent.

### Phylomorphospace occupation

**Figure 5:**
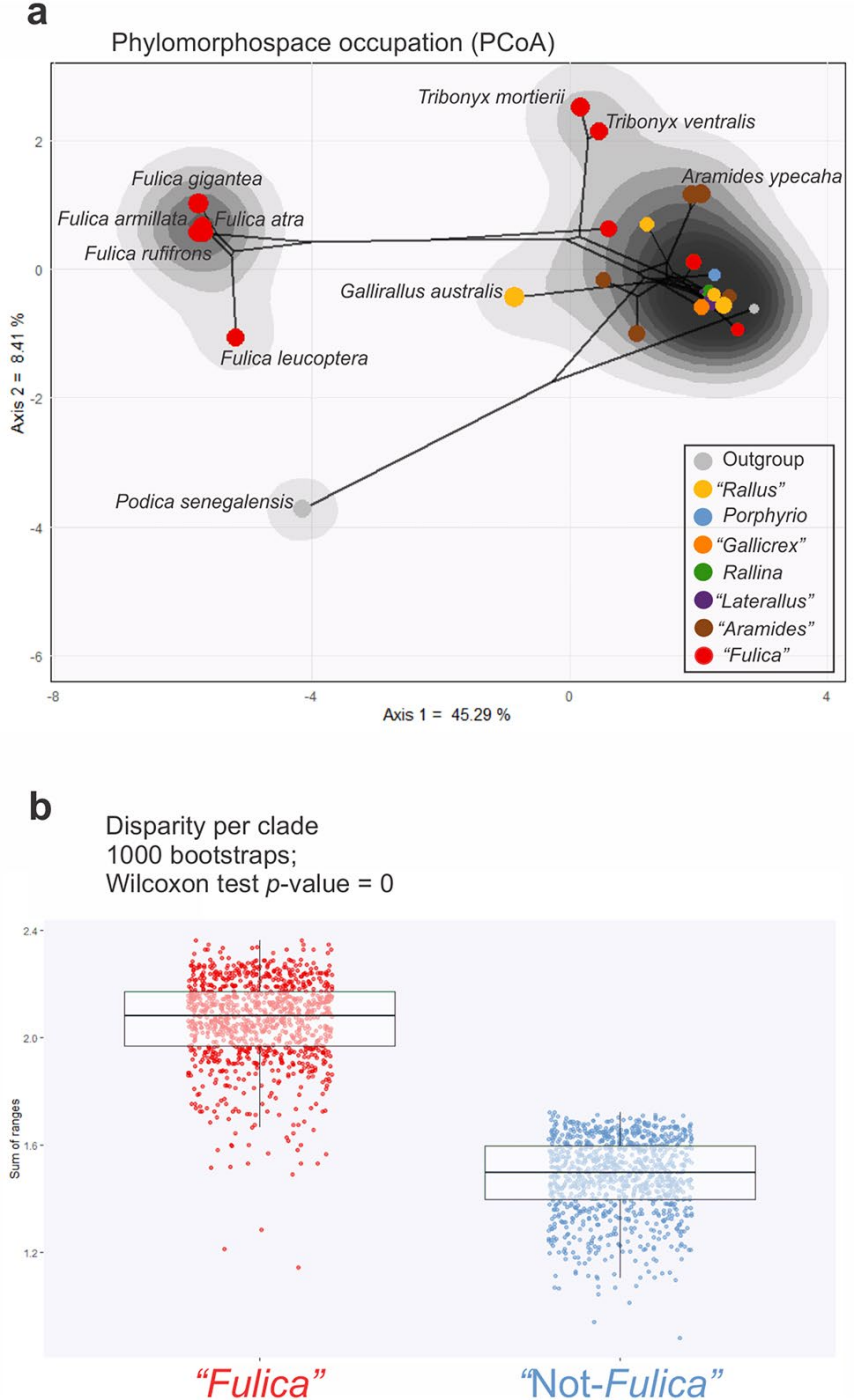
(**a**) Phylomorphospace occupation of the PCoA, dots colored according to its belonging to clade within Rallidae (see Fig. 3a). (**b**) Comparison of disparity between the clade “*Fulica*” and the rest of Rallidae, labeled as “non-*Fulica*”.

The phylomorphospace reveals a major separation between those in the genus *Fulica* and the rest of the species, along the first axis (Fig. 5a). Among the outgroup taxa, *Podica* (Heliornithidae) is separated from the rest of the species along the two first axes, but *Sarothrura* (Sarothruridae) is clustered within the rallids (besides *Fulica*).

The main cluster of the rallids is in the positive values of the first axis, with many genera clustered in the region of the highest density of phenotypes, which also includes the greater richness of clades (as revealed by the variation of colors of the dots). The regions within this cluster are occupied, from higher to lower density, in the following order: *Rallus aquaticus* (with other species of *Rallus* located in the region with the highest density), *Aramides* and *Pardirallus* (both belonging to the "*Aramides*" clade, with another member of this clade, *Porzana albicollis*, situated in the region of highest density). Finally, *Gallirallus australis* and most members of the "*Fulica*" clade (excluding *Fulica* and *Porzana porzana*, the latter located in the region of highest density) occupy the least dense regions of this cluster. The five analysed species of *Fulica* form a tight cluster in the negative region of the first axis and the positive region of the second axis. Only *F*. *leucoptera* is slightly separated from the rest, positioned along the negative values of the second axis.

From the clades within rallids, “*Fulica*” is the one that occupies the largest portion of the phylomorphospace. This extends from *Porzana porzana* in the region of the highest density of phenotypes to the two species of *Tribonyx* on the rim of the larger cluster; and onto the other cluster comprising the species of *Fulica*, with no intermediate phenotypes.

### Disparity analysis

The disparity within the clade "*Fulica*", which includes *Fulica*, *Gallinula*, *Tribonyx*, *Porphyriops*, and *Porzana porzana*, is approximately equivalent to that of the rest of the rallids (Fig. 5b). Despite that the other genera besides *Fulica* within the clade “*Fulica*” are similar to the rest of the rallids, the Wilcoxon test clearly indicates that the "*Fulica*" clade is significantly disparate from the rest of the rallids (*p*-value = 0).

## Discussion

Surface swimming is common in many aquatic birds, including ducks, cormorants, loons, grebes, gulls, pelicans, gannets, phalaropes, among others^1^. Many of these birds are also extremely adapted for foot-propelled diving, like loons, cormorants, grebes, and many ducks^1,2^. While the morphology of extreme foot-propelled divers is well-studied^1,2,18,20,28,29^, the morphology in more generalist taxa that can also dive have been poorly studied.

Rallidae is a well-suited clade for exploring the origin of swimming and diving adaptation, as in this family there is a spectrum from terrestrial to swimming and diving taxa^11,15,30^. In this study, we analysed the variation of postcranial osteological characters typically considered adaptive to the diving habit in different rallids in a comparative context. Our exploration ranged from anatomical descriptions and morphometric techniques, to the construction of a phylomorphospace and a disparity analysis considering ecomorphologically relevant characters.

Many osteological traits considered adaptations to diving are absent in *Fulica*, the most diving rallid. This is true for the femur (such as the absence of the trochanter femoris and the strong marked curvature), and the tarsometatarsus (such as the torsion along the main axis^29^). The relative proximal extension of the head of the femur and the trochanter femoris (which delimit the facies articularis antitrochanterica that articulates with the antitrochanter of the pelvis) determines the angle at which the femur separates from the pelvis^22^. Therefore, this morphology is associated with the abduction of the leg and feet during diving^22^. In cormorants and darters, the femur is curved but the head and the trochanter show similar proximal extension^23^. During diving, cormorants kept the feet below the body and the femur is not abducted^10^. However, other traits related to diving are present in of *Fulica.* This is true for the pelvis, in which the inter-acetabular and post-acetabular regions are compressed, a feature observed in diving ducks^20^. The post-acetabular region is approximately the same size as the pre-acetabular region^15^, another trait also shared with diving ducks^20^. In terrestrial rallids on the contrary, the preacetabular region is longer; while in diving taxa the postacetabular region is markedly longer^5^. This is demonstrated in the gradient of increasing adaptation to diving observed among *Anas*, *Heteronetta*, *Oxyura*, and *Biziura*^20^.

Diving birds extend the knee region in different ways through the participation of the patella, the proximal region of the tibiotarsus and the associated muscles and tendons^20,28^. The extinct *Hesperornis*, like the extant cormorants and diving ducks have a large and robust patella that is attached to a slightly proximally extended crista cnemialis cranialis^28,35^. In grebes, both the patella and the crista cnemialis cranialis are proximally extended^28^. However, in loons the patella is extremely reduced, being embedded in the patellar tendon^28^, and only the crista cnemialis cranialis is highly extended. *Fulica* shows a slightly proximally extended crista cnemialis cranialis but other rallids do not exhibit this morphology.

A narrow and streamlined body is characteristic of diving birds, for either propelled by feet or by wings^2–4^. This trait is also found in non-avian swimming taxa, such as mosasaurs^36^ and the platypus^37^. Birds achieve this diver anatomy by having the vertebral section of their ribs oriented at a low angle with their spine^3,4^. In foot-propelled diving birds, the sternum is narrow, especially in the middle part, but this characteristic is also found in some terrestrial birds^38^. *Fulica*, along with closely related taxa included in the clade “*Fulica*” within rallids, have broader sternums than other rallids. This may be related to buoyant swimming, while in other rallids it is especially compressed, likely an adaptation to moving in dense vegetation^39^. This, along with the fact that most characters in the genus *Fulica* recovered herein and related to diving—such as the proximal prolongation of the crista cnemialis cranialis, the elongation of the postacetabular region of the pelvis, and the increase of the femoral splay angle—are noticeable only in comparison with other rallids. This reinforces the clade-dependent nature of early adaptation or adaptation without specialization.

Greater femoral splay angles are found in swimming birds^22^, especially in grebes (Podicipedidae) and loons (Gaviidae). This would explain the greater values in *Fulica* compared with the other rallids and also would explain the difference found between *Fulica armillata* and *F*. *rufifrons*, as the latter feed primarily from surface vegetation^40^ while the former feeds mostly by diving^16^.

In our analysis that considers ecomorphologically relevant characters related to swimming and diving in birds, the clade “*Fulica*” is significantly disparate from other rallids. While many features of specialized foot-propelled divers are present in *Fulica*, particularly in the tibiotarsus and the pelvis, these features are only noticeable when compared with other rallids, as the degree of development of these features is markedly lesser than in loons, grebes, or cormorants. This could be indicating a stabilizing selection of characteristics associated with a generalist morphology in *Fulica*, their generalist habits^11^ such as perching, walking and running, and a body structure where the legs are placed in the center of the body instead of moving caudally as in extreme divers^5^.

This agrees with the concept that the study of ecomorphology and adaptation benefits from a phylogenetically informed perspective^41^. Conversely, studying adaptation in generalist taxa broadens our understanding of ecomorphologically significant characteristics, thereby enabling us to generalize their evolutionary patterns.

## Methods

### Taxa selection and osteological comparisons

Postcranial bones of four species of *Fulica* (*F. leucoptera*, *F. gigantea*, *F. armillata*, and *F. rufifrons*) were compared with other species of the family Rallidae (*Aramides cajaneus*, *A. ypecaha*, *Gallinula chloropus*, *Porphyriops melanops*, *Laterallus melanophaius*, *Pardirallus maculatus*, *Pa. sanguinolentus*, *Porphyrio martinica*, *Porzana albicollis*, *Porz*. *flaviventer*, *Rallus limicola*, and *R*. *longirostris*) (see Supplementary Information S1 for further details of the specimens). Skeletal micro tomography scans of the rallids *Fulica atra*, *Tribonyx mortierii*, *T. ventralis*, *Porzana porzana*, *Rallina eurizonoides*, *Amaurornis phoenicurus*, *Gallirallus australis* and *Rallus aquaticus*, and from the *Podica senegalensis* (Heliornithidae) and *Sarothrura rufa* (Sarothruridae) were downloaded from MorphoSource, rendered in 3D using the module SlicerMorph^42^ of the Open Source software 3DSlicer 5.4.0 (slicer.org^43^), and used for comparisons. Characters associated to foot-propelled diving (see Supplementary Information S2) were recovered from the literature (e.g., Dabelow^2^; Frank and Neu^44^; Raikow^20^; Worthy and Lee^21^; Zelenkov^45^). Identification of analysed specimens is provided in Supplementary Information S1. Anatomical nomenclature follows Baumel and Witmer^46^.

### Phylogenetic background

The phylogenetic tree used in the phylogenetically informed approaches in this study was obtained from García-R et al.^47^. The tree was selected because it is a time-calibrated molecular phylogeny containing all the species analysed in this study (Fig. 3a). The subclades within Rallidae are represented in the tree, following García-R et al.^47^. The Gruiformes *Podica senegalensis* (Heliornithidae), and *Sarothrura rufa* (Sarothruridae) were selected as outgroup.

### Measurements

All bones were photographed with the optic axis orthogonal to their longest axis. Linear and angular measurements were obtained using ImageJ^48^.

#### Hind limb linear measurements

We evaluated the proportions of hind limb elements of *Fulica* species compared to different rallids by measuring the length of the femur (from the fascies articularis antitrochanterica to the condyles), tibiotarsus (from the area interarticularis to the condyles), and tarsometatarsus (from the cotylae to the trochlea metatarsi III) (Fig. 3b). The linear measurements were plotted on a ternary plot, where each vertex of the equilateral triangle represents an impossible 100% of each bone (Fig. 4c). The ratios of the three variables in different individuals are plotted as positions within that triangle. The individuals are plotted as dots colored according to the clade that they belong (see Phylogenetic background section). Individuals of *Fulica* are differentiated from individuals in other genera within the clade “*Fulica*”. The ternary plot was generated using the package Ternary^49^ of the programming language R^50^.

#### Femoral splay angle

We measured the femoral splay angle^22^ (i.e., the angle of abduction of the femur from the midline of the synsacrum) by holding the femur and pelvis in a horizontal position and with the femur abducted, as the antitrochanter of the pelvis articulates with the facies articularis antitrochanterica of the femur. Then, we traced a line along the axis of the femur crossing the lateral condyle and trochanter femoris, and finally we measured the angle of intersection between this line and the midline of the synsacrum (Fig. 3c). We mapped the average for each species in the dated molecular phylogeny^47^ using the method of maximum likelihood (Fig. 4b). The character mapping was made using the package phytools^51^ of the programing language R^50^.

### Ordination methods and phylomorphospace occupation

We utilized a phylomorphospace to visualize the density of traits associated with foot-propelled diving and the macroevolutionary landscape of rallids. This was done by adapting the protocol established by Fischer et al.^52^ and Laboury et al.^53^. To achieve this, we created a character matrix that included a codification of the ecomorphologically informative qualitative characters previously mentioned (Characters 1–15; Figs. 1, 2), the proportion between femur and tibiotarsus, the proportion between tarsometatarsus and tibiotarsus, and the femoral splay angle for all the analysed rallids.

Each continuous trait was normalized prior to the analyses using the built-in R function scale. The character matrix was transformed into a distance matrix using the Gower distance metric, which allows for the calculation of dissimilarity between different species using both binary and continuous data. The Gower distance matrix was converted into a phylomorphospace through a principal coordinates analysis (PCoA) with the Cailliez correction for negative eigenvalues, implemented in the ape package^54^ (Fig. 5a). The density of phylomorphospace occupation was estimated using the ggphylomorphospace function of Barr^55^ as modified by Fischer et al.^52^. All analyses were performed in R^50^.

### Morphofunctional disparity analysis

We used all the axes of the PCoA to perform a disparity analysis of rallids (Fig. 5b), based on the ecomorphological dataset. We categorized the rallids into two groups: those in the “*Fulica*” clade (which, in our dataset, includes the genera *Fulica*, *Tribonyx*, *Gallinula*, and *Porzana porzana*), and the remaining rallids, labeled as “Not-*Fulica*” (refer to the phylogenetic background section and Fig. 3a). We made this division to create two groups of comparable size. It's also worth noting that the “*Fulica*” clade comprises more aquatic genera (particularly the species of *Fulica*) than the other rallids.

The disparity distribution for each group, “*Fulica*” and “Not-*Fulica*”, was computed using a bootstrapping method with 1000 iterations. This computation was performed using the dispRity function from the dispRity package^56^. We evaluated the significance of the disparity difference between the two groups using a Wilcoxon test (a non-parametric alternative to the Student's *t*-test). The disparity test was conducted using the test.dispRity function from the dispRity package^56^. The entire procedure was executed in the R programming language^50^.

### Supplementary Information

Supplementary Information 1.

Supplementary Information 2.

## Data Availability

Raw measurements are provided in Supplementary Information.
