# Supplementary material for: Specialized diving traits in the generalist morphology of Fulica (Aves, Rallidae)
Source: Sci Rep. 2024 Jun 17;14:13966. doi: 10.1038/s41598-024-64853-4 (PMC11183161; doi:10.1038/s41598-024-64853-4)
Supplement: Supplementary file 2 — Supplementary Information 2. [file 41598_2024_64853_MOESM2_ESM.docx]

**Specialized diving traits in the generalist morphology of *Fulica* (Aves, Rallidae)**

Ricardo S. De Mendoza^1^*, Julieta Carril^1^, Federico J. Degrange^2^, and Claudia P.

Tambussi^2^

^1^Laboratorio de Histología y Embriología Descriptiva, Experimental y Comparada

(LHYEDEC), Facultad de Ciencias Veterinarias, Universidad Nacional de La Plata,

Consejo Nacional de Investigaciones Científicas y Técnicas

(CONICET), Buenos Aires, Argentina.

^2^Centro de Investigaciones en Ciencias de la Tierra (CICTERRA), Universidad

Nacional de Córdoba, Consejo Nacional de Investigaciones Científicas y Técnicas

(CONICET), Córdoba, Argentina.

*Corresponding author: [rsdemendoza@gmail.com](mailto:rsdemendoza@gmail.com)

ORCID iDs:

RSDM: https://orcid.org/0000-0002-9642-1736

JC: https://orcid.org/0000-0001-8491-1677

FJD: https://orcid.org/0000-0002-9463-4893

CPT: https://orcid.org/0000-0002-8711-0549

Supplementary Information S2, character list:

1) Sternum, width: broad (0), narrow (1). The sternum of rallids is narrower than that of most birds, a characteristic typical of diving birds (Tickle et al., 2007; Lee et al., 2022). Contrary to most rallids, the sternum of *Gallinula* and *Fulica* is broad. This character aims to capture the differences between these two distinct morphologies, thus it was treated as discrete.

2) Ribs, orientation with spine: angle of the ribs with the spine close to 90 degrees (0), smaller angle (1). Many diving animals, in order to achieve a more streamlined body, have narrow angles between the ribs and the spine (e.g., Lindgren et al., 2010). Diving birds are no exception to this (Tickle et al., 2007). However, rallids, have a narrow body while not being diving birds. An exception to this is seen among the most aquatic genera, as in character 1.

3) Pelvis, post-acetabular region in dorsal view: wide (0), compressed (1). Diving birds have compressed pelvis, especially in the post-acetabular region, becoming wider only in the caudal-most part (Raikow, 1970; Ibáñez and Tambussi, 2012).

4) Pelvis, caudal dorsoventral depth: same dorsoventral depth as the cranial parts of the pelvis (0), larger dorsoventral size in caudal-most part of the pelvis (1). In diving birds, the caudal-most part of the pelvis is approximately the same dorsoventral size as the cranial parts. In contrast, non-diving birds typically have a dorsoventrally larger caudal-most part of the pelvis (see Ibáñez and Tambussi, 2012; Fig. 2).

5) Pelvis, enlarged antitrochanter: no (0), yes (1). Diving birds exhibit an enlarged antitrochanter (Clifton et al., 2017).

6) Pelvis, length of post-acetabular region: clearly shorter than pre-acetabular region (0), same size or larger than pre-acetabular region (1). Diving birds have a longer post-acetabular region of the pelvis (Raikow, 1970; Ibáñez and Tambussi, 2012).

7) Femur, robustness: thin (0), stout (1). Diving birds usually exhibit short and stout femora (Townsend, 1909; Raikow, 1970; Noriega et al., 2008; Clifton et al., 2017).

8) Femur, craniocaudal curvature: shaft straight (0), curved, especially the distal third of the shaft (1). A craniocaudally curved femur is a typical character of diving birds (Noriega et al., 2008). On the other hand, the femur is typically curved in rallids. Therefore, in this analysis, this character differentiates rallids from the outgroup taxa.

9) Femur, sulcus patellaris: narrow, not well defined (0), well defined, straight edges, broad (1). A well-marked sulcus patellaris is characteristic of diving birds.

10) Tibiotarsus, crista cnemialis cranialis, proximal extension: not proximally extended, about the same level of the facies articularis (0), proximally extended, in medial or lateral view the crista exhibits a well-defined curvature followed by a proximal development stronger than in 0 (1). A proximally enlarged crista cnemialis cranialis is one of the best-known osteological features of diving birds (Shufeldt, 1904; Raikow, 1970; Noriega et al., 2008; Worthy and Lee, 2008; Bell et al., 2019, Segesdi and Pecsics, 2022).

11) Tibiotarsus, facies cranialis: round (0), flat (1). In foot-propelled diving birds the tibiotarsus is usually flat. This characteristic could be restricted to the level of the crista fibularis as observed in diving ducks (Worthy and Lee, 2008; De Mendoza and Tambussi, 2019), or it could extend to near the condyles, as seen in grebes (Shufeldt, 1904).

12) Tibiotarsus, distal end, condyles medially displaced: no, condyles aligned with the distal end of the shaft, or even condylus lateralis laterally displaced (0), yes, condyles medially displaced, with condylus lateralis aligned with sulcus m. fibularis (1). In buoyant surface swimming and in foot propelled diving taxa, the condyles are usually displaced medially.

13) Tibiotarsus, distal end, large condylus medialis: no (0), yes (1). A large, sometimes hooked condylus medialis is a common feature of foot-propelled diving birds (Worthy and Lee, 2008; De Mendoza and Tambussi, 2019).

14) Tarsometatarsus, proximal end, large eminentia intercotylaris: no (0), yes, proximally protruding and pointed (1). Foot-propelled buoyant swimmers and divers exhibit a reduced and blunt eminentia intercotylaris in the tarsometatarsus. This feature enables an increase in the range of motion of the tibiotarsa-tarsometatarsal joint (Zelenkov, 2020).

15) Tarsometatarsus, tapering distal to cotylae: no (0), yes (1). In some foot-propelled buoyant swimmers and divers the cotylae, especially the cotyla medialis, form a large cup, while the surface immediately below is smaller. This in dorsal aspect is seen like a tapering from the most-proximal part of the bone towards the shaft (Noriega et al., 2008; Worthy and Lee, 2008).

16) Hind limb, ratio between the femur and tibiotarsus (femur length/tibiotarsus length). A character typical of diving birds is an especially short femur and an especially long tibiotarsus (Shufeldt, 1904; Raikow, 1970; Nudds et al., 2012; Bell et al., 2019; Bell and Chiappe, 2022). As the variation of this character is not clearly captured into discrete morphotypes, the character was treated as continuous.

17) Hind limb, ratio between the tarsometatarsus and tibiotarsus (tarsometatarsus length/tibiotarsus length). Besides a long tibiotarsus, many diving birds exhibit a short tarsometatarsus (Raikow, 1970; Nudds et al., 2012; Clifton et al., 2017). As the variation of this character is not clearly captured into discrete morphotypes, the character was treated as continuous.

18) Femoral splay angle. Angle of abduction of the femur from the midline of the synsacrum (see Methods section and Fig. 1 in main text).

**References**

Bell, A., & Chiappe, L. M. (2022). The Hesperornithiformes: A Review of the Diversity, Distribution, and Ecology of the Earliest Diving Birds. *Diversity*, 14(4), 267; https://doi.org/10.3390/d14040267

Bell, A., Yun-Hsin, W., & Chiappe. L. M. (2019). Morphometric comparison of the Hesperornithiformes and modern diving birds. *Palaeogeography, palaeoclimatology, palaeocology* 513:196-207

Clifton, G. T., Carr, J. A., & Biewener, A. A. (2017). Comparative hindlimb myology of foot-propelled swimming birds. *Journal of Anatomy*, 232, 105–123.

De Mendoza, R. S., & Tambussi, C. P. (2019). *Cayaoa bruneti* (Aves: Anseriformes) from the early Miocene of Patagonia, Argentina: New materials and revised diagnosis. *Ameghiniana*, 56, 213.

Ibañez, B., & Tambussi, C. P. (2012). Foot-propelled aquatic birds: Pelvic morphology and locomotor performance. *Italian Journal of Zoology*, 79, 356–362.

Lee S., Lee, Y.-N., Currie, P. J., Sissons, R., Park, J.-Y., Kim, S.-H., Barsbold, R. & Tsgtbaatar, K. (2022). A non-avian dinosaur with a streamlined body exhibits potential adaptations for swimming. *Communications Biology*, 5: 1185.

Noriega, J. I., Tambussi, C. P., & Cozzuol, M. A. (2008). New material of *Cayaoa* *bruneti* Tonni, an early Miocene anseriform (Aves) from Patagonia, Argentina. *Neues Jahrbuch für Geologie und Paläontologie*, 249, 271–280.

Nudds, R. L., Atterholt, J., Wang, X., You H.-L., & Dyke, G. L. (2012). Locomotory abilities and habitat of the Cretaceous bird *Gansus yumenensis* inferred from limb length proportions. *Journal of Evolutionary Biology*, 26: 150-154. https://doi.org/10.1111/jeb.12036

Raikow, R. J. (1970). Evolution of diving adaptations in the stifftail ducks. *University of California Publications in Zoology*, 94: 1–51.

Segesdi, M., & Pecsics, T. (2022). Trends of avian locomotion in water - an overview of swimming styles. *Ornis Hungarica*. 30. 30-46. 10.2478/orhu-2022-0003.

Shufeldt, R. W. (1904). An arrangement of the families and the higher groups of birds. *Amer. Nat.*, 38:833–857

Tickle, P. G., Ennos, A. R., Lennox, L. E., Perry, S. F., & Codd, J. R. (2007). Functional significance of the uncinate processes in birds. *J Exp Biol*, 210 (22): 3955–3961.

Townsend, C. W. (1909). The use of the wings and feet by diving birds. *The Auk*, 26, 234–248.

Worthy, T. H., & Lee, M. S. Y. (2008). Affinieties of Miocene waterfowl (Anatidae: *Manuherikia*, *Dunstanetta* and *Miotadorna*) from the St Bathans fauna, New Zealand. *Palaeontology*, 51, 677–708.

Zelenkov, N. (2020). The oldest diving anseriform bird from the late Eocene of Kazakhstan and the evolution of aquatic adaptations in the intertarsal joint of waterfowl. Acta Palaeontologica Polonica, 65, 65.
